# Probing Intersubunit Interfaces in AMPA-subtype Ionotropic Glutamate Receptors

**DOI:** 10.1038/srep19082

**Published:** 2016-01-07

**Authors:** Maria V. Yelshanskaya, Kei Saotome, Appu K. Singh, Alexander I. Sobolevsky

**Affiliations:** 1 Department of Biochemistry and Molecular Biophysics, Columbia University 650 West 168th Street, New York, NY 10032.

## Abstract

AMPA subtype ionotropic glutamate receptors (iGluRs) mediate the majority of fast neurotransmission across excitatory synapses in the central nervous system. Each AMPA receptor is composed of four multi-domain subunits that are organized into layers of two amino-terminal domain (ATD) dimers, two ligand-binding domain (LBD) dimers, transmembrane domains and carboxy-terminal domains. We introduced cysteine substitutions at the intersubunit interfaces of AMPA receptor subunit GluA2 and confirmed substituted cysteine crosslink formation by SDS-PAGE. The functional consequence of intersubunit crosslinks was assessed by recording GluA2-mediated currents in reducing and non-reducing conditions. Strong redox-dependent changes in GluA2-mediated currents were observed for cysteine substitutions at the LBD dimer-dimer interface but not at the ATD dimer-dimer interface. We conclude that during gating, LBD dimers undergo significant relative displacement, while ATD dimers either maintain their relative positioning, or their relative displacement has no appreciable effect on AMPA receptor function.

Ionotropic glutamate receptors (iGluRs) are a family of tetrameric ligand-gated ion channels that mediate the majority of excitatory neurotransmission in the central nervous system and are implicated in numerous devastating neurological diseases^1^,^2^,^3^. While the family includes members with distinct biophysical and pharmacological properties, such as NMDA, AMPA and kainate receptors, each iGluR subunit has a conserved modular design that is comprised of ATD, LBD, ion channel and CTD. Domain interaction by virtue of intersubunit interfaces plays a crucial role in iGluR assembly^4^,^5^,^6^,^7^,^8^. Intersubunit interfaces are also involved in iGluR gating and contribution of the LBD intradimer interface in particular has been the subject of thorough investigation^9^,^10^,^11^,^12^,^13^,^14^,^15^,^16^. The role of other intersubunit interfaces is less understood. For example, the ATD dimer-dimer interface appears prominent in different isolated ATD^17^,^18^,^19^,^20^,^21^ and full-length iGluR crystal structures^5^,^16^,^22^,^23^, consistent with a purely structural role in non-NMDA receptor assembly. However, recent structural studies suggest that AMPA receptor desensitization disrupts the ATD dimer-dimer interface and the two ATD dimers separate from each other by tens of angstroms^22^,^24^,^25^. On the other hand, such significant separation does not appear to be critical for desensitization because complete removal of ATD domains results in only small changes in the functional characteristics of desensitization^26^. In this study, we probed the relative positions of iGluR domains during gating by evaluating the functional consequence of introducing cysteine crosslinks at the interdomain interfaces along the axis of overall two-fold rotational symmetry (Fig. 1).

## Results

**Figure 1:**
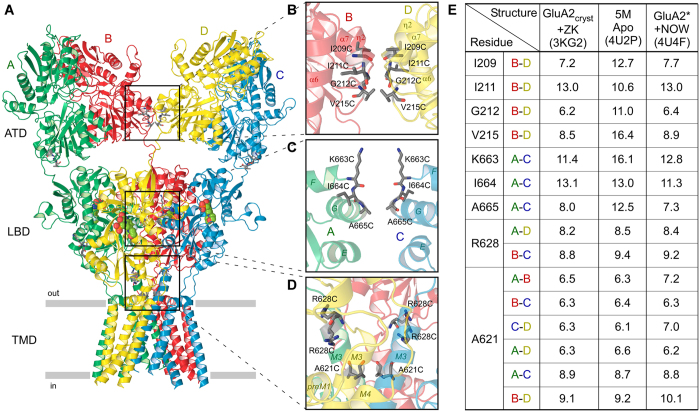
Location of substituted cysteines and distances between them. (**A**) Ribbon diagram of the GluA2_cryst_ structure in complex with competitive antagonist ZK200775 (PDB ID: 3KG2). Four subunits (**A**–**D**) are in different colors. (**B**–**D)** Close-up views of intersubunit interfaces between two ATD dimers (**B**), two LBD dimers (**C**) and LBD-TMD linkers and ion channel domains (**D**). Residues substituted with cysteines are shown as sticks. The ribbon diagram is semitransparent. In c, the subunits B and D are removed for clarity. (**E)** Table showing distances (in Å) between Cα’s of residues substituted with cysteines and measured in three selected structures: GluA2_cryst_ in complex with ZK200775 (PDB ID: 3KG2), 5M construct in the apo state (PDB ID: 4U2P) and GluA2* in complex with partial agonist NOW (PDB ID: 4U4F). For R628 and A621, the distances are shown for more than one pair of residues that belong to different pairs of subunits.

We introduced nine mutations at or near the intersubunit interfaces of rat GluA2i AMPA-subtype iGluR (Fig. 1A): four cysteine substitutions at the ATD dimer-dimer interface (I209C, I211C, G212C and V215C; Fig. 1B), three cysteine substitutions at the LBD dimer-dimer interface (K663C, I664C and A665C; Fig. 1C), one cysteine substitution at the LBD-TMD linker region (R628C; Fig. 1D) and one cysteine substitution in the ion channel (A621C; Fig. 1D). The distances between Cα’s of the cysteine-substituted residues in selected published crystal structures of GluA2 (Fig. 1E) suggest the possibility of cysteine crosslinking, at least in certain activation states of the receptor.

**Figure 2:**
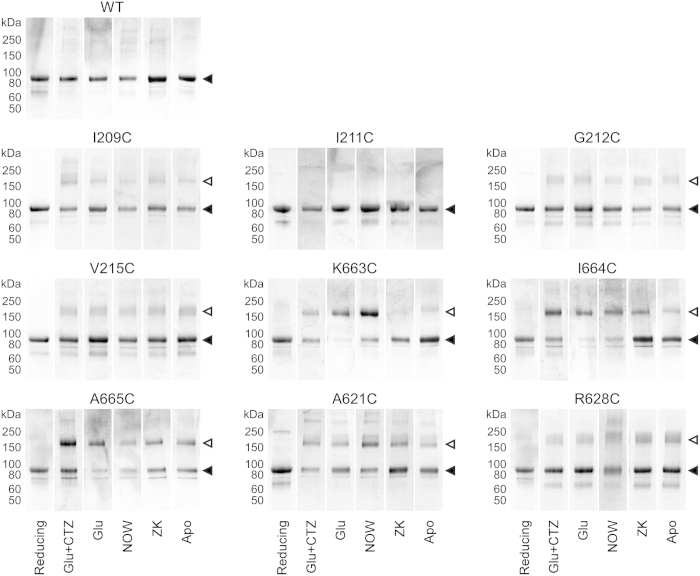
SDS-PAGE analysis of substituted cysteine crosslinking. Experiments were carried out with wild type (WT) or cysteine-substituted GluA2_C589A-Thr_ receptors either in reducing conditions (2 mM DTT) or in non-reducing conditions but in the presence of 3 mM Glu and 50 μM CTZ (Glu + CTZ, favoring the open state) or 3 mM Glu (Glu, favoring the desensitized state) or 500 μM NOW (NOW, favoring the desensitized state) or 100 μM ZK (ZK, favoring the antagonist bound closed state) or in the absence of ligands (Apo, favoring the unliganded closed state). For I209C and A621C, Cu:Phen was added to the non-reducing dialysis buffers (see Methods). Filled and open triangles indicate positions of monomeric and dimeric bands, respectively.

To test substituted cysteine crosslinking, we purified full-length GluA2 receptors in reducing conditions and dialyzed the protein into non-reducing buffers in the presence of ligands favoring different activation states (Fig. 2, Supplementary Fig. 1). All receptors in these experiments had C589A substitution in the M2 loop to prevent non-specific protein aggregation^5^, as well as extra residues from the thrombin cleavage site (GLVPR) at the distal C-terminus required for protein purification (see Methods). The resulting construct GluA2_C589A-Thr_ eluted as a single major peak from the size-exclusion column (Supplementary Fig. 1), was represented by a single band on SDS-PAGE in both reducing and non-reducing conditions (Fig. 2) and had functional properties indistinguishable from wild type GluA2 receptors (Supplementary Tables 1–3).

The apparent crosslinking propensity of cysteines introduced at the interdomain interfaces was revealed by appearance of higher molecular weight bands corresponding to dimer formation on SDS-PAGE in non-reducing conditions (Fig. 2). Several introduced cysteines showed dependence of crosslinking on the activation state of receptor. For example, K663C and I664C showed a greater propensity to crosslink in the presence of full or partial agonists than in the absence of ligands or in the presence of the competitive antagonist ZK 200775. This pattern of crosslinking suggests that the corresponding residues are more accessible for crosslinking in desensitized states than in closed states. For several cysteines (I209C, G212C, V215C and R628C), the dimeric bands looked weaker than the monomeric bands, possibly due to non-ideal relative positioning of substituted residues and the rigidity of environment (the ATD dimer-dimer interface). Cysteines substituting isoleucines I211, which have side chains facing away from each other (Fig. 1B), did not appear to crosslink in any conditions. Notably, FSEC analysis showed that each of the crosslink mutants retain the native tetrameric state in the various tested conditions (Supplementary Fig. 1). Taken together, these observations suggest that the majority of the substituted cysteines can crosslink in the context of physiologically relevant receptor conformations.

**Figure 3:**
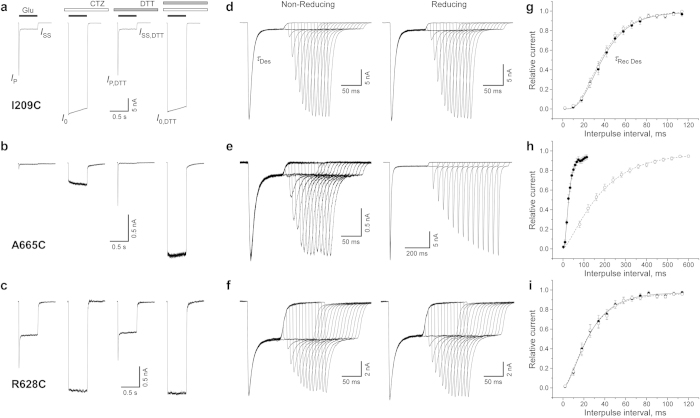
Electrophysiological recordings from cysteine-substituted GluA2 receptors. Examples are shown for cysteine substitutions at the interfaces between two ATD dimers (I209C: a, d and g), two LBD dimers (A665C: b, e and h) and at the top of the ion channel (R628C: c, f and i). (**a–c**) Representative whole-cell currents recorded at –60 mV membrane potential from HEK293 cells expressing cysteine-substituted receptors in response to a 500 ms application of 1 mM Glu alone and applications of Glu in the continuous presence of 30 μM CTZ, 2 mM DTT, or both CTZ and DTT. (**d–f**) Currents recorded using a two-pulse protocol in the absence (non-reducing condition, left) or presence (reducing condition, right) of 1 mM DTT, in which an initial application of 3 mM Glu was made to produce steady-state desensitization and was repeated after allowing the channels to recover from desensitization for different length of time. The envelope of the peak currents evoked by the series of the second applications gives the time course of recovery from desensitization^54^. (**g–i**) Mean recovery from desensitization measured in non-reducing (filled circles) and reducing (open circles) conditions using the protocols illustrated in d-f. Straight and dashed curves are fits with Equation 1 (see Methods) for non-reducing and reducing conditions, respectively. The values of the fitting parameters are listed in Supplementary Table 3. Errors are SEM.

**Figure 4:**
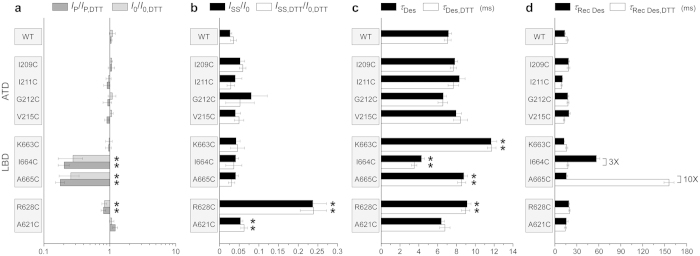
Effects of cysteine substitutions on gating. (**a**) Effects on activation. Shown are the ratios of the peak currents amplitudes (*I*_P_/*I*_P,DTT_) and the maximal current amplitudes in the continuous presence of CTZ (*I*_0_/*I*_0,DTT_) measured in the absence and presence of DTT. (**b–d)** Effects on desensitization. Shown are the fraction of non-desensitized receptors (**b**), the time constant of desensitization (**c**) and the time constant of recovery from desensitization (**d**) measured in the absence (filled bars) or presence (open bars) of DTT. Asterisks indicate values significantly different from 1 (**a**) or from the wild type values (**b,c**); t-Tests (p < 0.05). Errors are SEM.

To study the effects of substituted cysteines on channel gating, we expressed wild type and cysteine-substituted GluA2 receptors in HEK293 cells and used patch-clamp techniques with fast solution exchange to record GluA2-mediated currents. At –60 mV, glutamate application elicited a typical AMPA receptor response: an inward current that quickly decayed in the continuous presence of glutamate as a result of desensitization (Fig. 3a–c). Desensitization was blocked by the positive allosteric modulator cyclothiazide (CTZ). Independent of the presence of CTZ, most cysteine-substituted mutants (e.g., I209C in Fig. 3a and R628C in Fig. 3c) and wild type receptors showed similar responses to 0.5-s applications of glutamate in reducing and non-reducing conditions (Supplementary Table 1, Fig. 4a). However, two cysteine substitutions at the LBD dimer-dimer interface, I664C and A665C (Fig. 3b), showed significantly smaller currents in non-reducing compared to reducing conditions, consistent with previous studies^16^,^27^,^28^. The ratio of the maximal current amplitudes in non-reducing versus reducing conditions in the presence of CTZ (*I*_0_/*I*_0,DTT_) or its absence (*I*_P_/*I*_P,DTT_) were 0.28 ± 0.11 (n = 7) or 0.20 ± 0.04 (n = 12) for I664C and 0.26 ± 0.09 (n = 7) or 0.18 ± 0.03 (n = 25) for A665C, respectively. The straightforward interpretation of the current reduction in non-reducing conditions is that crosslinking of these cysteines prevented activation of GluA2 receptors. Small but statistically significant current reduction in non-reducing conditions (*I*_0_/*I*_0,DTT_ = 0.83 ± 0.07, n = 25 and *I*_P_/*I*_P,DTT_ = 0.80 ± 0.07, n = 25) was also observed for R628C substitution in the M3-S2 linker. Previously, potentiation of R628C-mediated currents was reported in response to application of the MTS reagent MTSET in the presence of glutamate^29^.

To quantify the effects of cysteine substitutions on GluA2 desensitization, we measured several parameters. We used the ratio of the steady state and maximal current amplitudes (*I*_SS_/*I*_0_ or *I*_SS,DTT_/*I*_0,DTT_, Fig. 3a) as an estimate for the fraction of non-desensitized channels (Supplementary Table 2, Fig. 4B). The majority of mutants had this parameter similar to wild type receptors. A significantly lower extent of desensitization was observed for R628C (Fig. 3c), a position where previous studies showed that glutamate substitution results in nearly complete block of desensitization^29^. This weakening of desensitization was redox-independent, suggesting an electrostatic and/or steric effect of this mutation on GluA2 desensitization^29^,^30^. Small but significant reduction of desensitization was also detected for the A621C mutant, reminiscent of a stronger effect on desensitization previously observed for the A621G mutant^31^. A621 is a part of the highly conserved SYTANLAAF motif^32^ apparently involved in iGluR gating^31^,^33^,^34^,^35^,^36^,^37^.

We measured the rate of desensitization by fitting current decay in the continuous presence of glutamate (Fig. 3d). Compared to wild type, slower entry into desensitization was observed for K663C, A665C and R628C, while it was faster for I664C (Supplementary Table 2, Fig. 4C). Overall, changes in the time constant of desensitization were relatively small and redox-independent. For example, the largest redox-dependent difference in the rate of desensitization was observed for I664C, which had slightly faster desensitization in reducing (*τ*_Des,DTT_ = 3.5 ± 0.3 ms, n = 12) compared to non-reducing conditions (*τ*_Des_ = 4.3 ± 0.3 ms, n = 16), results that are consistent with previous observations^27^.

To quantify the rate of recovery from desensitization, we utilized two-pulse protocols illustrated in Fig. 3d–f. The majority of cysteine-substituted receptors displayed recovery from desensitization that was similar to wild type and essentially identical in reducing and non-reducing conditions (e.g., I209C in Fig. 3g and R628C in Fig. 3i; Supplementary Table 3). However, two cysteine substitutions at the LBD dimer-dimer interface, I664C and A665C (Fig. 3h), showed strong redox-dependence. For one of them, I664C, we have previously seen unique redox-dependent behaviour in the two-pulse protocol^16^. In the previous study however, we carried out our recordings differently. First, we were recording wild type-like recovery from desensitization in reducing conditions, followed by switching to non-reducing conditions and observing a biphasic recovery. Moreover, long exposures to glutamate in non-reducing conditions gradually reduced I664C-mediated currents in a use-dependent manner that was completely recovered by application of the reducing agent^16^. In the present study, recordings in non-reducing conditions were made from cells that had never been exposed to DTT before. In this case, the current amplitude was ~5 times smaller (Fig. 4a) and the recovery from desensitization was complete (Supplementary Table 2), but it was 3 times slower (*τ*_Rec Des_ = 56.7 ± 4.7 ms, n = 13) than in the presence of DTT (*τ*_Rec Des, DTT_ = 17.8 ± 1.3 ms, n = 16). After DTT application, we were able to completely restore the non-reducing condition behaviour only by applying the oxidizing agent copper(II):phenanthroline (2:50 μM; data not shown).

While the I664C redox-dependent behaviour is consistent with the putative cysteine crosslink preventing recovery from desensitization, the reducing agent had an opposite effect on the kinetics of A665C (Fig. 3e,h). In fact, the recovery from desensitization for A665C in non-reducing conditions (*τ*_Rec Des_ = 15.8 ± 1.9 ms, n = 6) was 10 times faster than in reducing conditions (*τ*_Rec Des, DTT_ = 156 ± 7 ms, n = 17). Apparently, the A665C mutation itself slowed down recovery from desensitization, while the putative crosslink between A665C cysteines restored recovery to the wild-type rate. A potential explanation for this behaviour is that in wild type receptors, the side chains of A665 need to get into close contact for the recovery from desensitization to occur. The bulkier cysteine side chains do not allow this close contact unless they form a disulphide bond. Detailed understanding of the mechanism of redox-dependent effect of A665C on GluA2 desensitization will require further experimentation.

## Discussion

Tetrameric assembly of AMPA receptors is mediated by five types of intersubunit interfaces, including large ATD and LBD intradimer interfaces, large transmembrane interfaces and smaller ATD and LBD interdimer interfaces^38^. The ATD intradimer interfaces underlie an important role of ATD in receptor assembly^4^,^39^,^40^ and comprise large contact areas between the upper and lower lobes^17^,^18^,^19^,^20^,^21^, which naturally restrict conformational freedom of individual ATD clamshells and reduce the potential of allosteric regulation of non-NMDA receptors via the ATD domains. There are, however, reports of conformational fluctuations in ATD domains^41^,^42^,^43^ as well as a possible role of ATD in AMPA receptor regulation by TARPs^44^, suggesting some allosteric capacity for this domain in addition to its established assembly function. The LBD intradimer interfaces are highly dynamic and undergo significant modification during desensitization^9^,^11^,^14^,^16^,^25^,^27^,^45^.

In this study, we probed the functional effect of crosslinking ATD and LBD interdimer interfaces located along the axis of overall two-fold rotational symmetry (Fig. 1). We also tested cysteine substitutions of two residues located near this axis, A621 and R628, which are close enough to form intersubunit crosslinks (Fig. 2) and have been previously shown to contribute to gating-related domain movements. Indeed, cysteine substitution at the pore-facing A621 resulted in state-dependent accessibility to MTS reagents^46^, while glutamate substitution of R628 led to near complete block of AMPA receptor desensitization^29^. We found that R628C weakly inhibited activation and slowed down desensitization, while both A621C and R628C reduced the extent of desensitization (Fig. 4). The weakness or absence of effects on activation for these two positions indicates that the observed crosslinking (Fig. 2) most likely involved cysteines substituted in the neighbouring rather than diagonal subunits (Fig. 1D). Additionally, redox-independence of the effects of A621C and R628C on gating suggests that cysteine crosslinking does not further alter the equilibrium between activation states of the receptor, which has already been changed by cysteine substitutions. This phenomenon might in part be due to the unstable character of the cysteine crosslinks, as was previously observed for the LBD intradimer interface mutations^16^,^27^, but requires further investigation. Overall, the results for R628C and A621C confirmed the sensitivity of our approach to the effects of cysteine substitutions on AMPA receptor gating.

We tested three substitutions in the LBD dimer-dimer interface: K663C, I664C and A665C. We found that I664C and A665C strongly suppressed activation, K663C and A665C slowed down desensitization, I664C increased the rate of desensitization, but neither of these effects were strongly redox-dependent (Fig. 4). In contrast, recovery from desensitization was drastically different in reducing and non-reducing conditions (Figs 3 and 4). Our results are consistent with previous results for K663C, I664C and A665C mutants^5^,^16^,^27^,^28^,^45^ but provide new details about the effects of cysteine substitutions and their crosslinks on receptor gating. For example, Lau *et al.*^28^ concluded that under reducing conditions, GluA2-A655C had similar activation and desensitization kinetics to wild-type GluA2 and that A665C substitution had limited functional impact on GluA2 receptors, while our more detailed measurements revealed significant differences in the rates of entry and recovery from desensitization in A665C and wild type receptors. Overall, our results strongly suggest that the LBD dimer-dimer interface undergoes significant rearrangements during AMPA receptor gating, and desensitization in particular.

The ATD dimer-dimer interface was probed at four locations: I209, I211, G212 and V215 (Fig. 1B). Since isoleucine I211 side chains were facing away from the interface, I211C did not show apparent crosslinking and served as a negative control. Cysteines substituted at I209, G212 and V215 did form crosslinks (Fig. 2). If the dissociation of the ATD dimers by tens of Angstroms, as suggested in the previous structural studies^22^,^24^,^25^, is functionally relevant, we would expect dramatic changes in the gating parameters measured in reducing and non-reducing conditions. Instead, all the parameters for the ATD dimer-dimer interface mutants were indistinguishable from the wild type parameters (Fig. 4) suggesting that restriction of possible relative movements of the ATD domains by cysteine crosslink has no appreciable effect on AMPA receptor function. We therefore conclude that if changes in this interface do happen during gating they either do not have functional consequences or these changes are much smaller than the recently reported dramatic rearrangements, which might be artefacts of cryo-EM sample preparation^22^,^24^,^25^ or crystal lattice distortions^22^.

In summary, we introduced cysteine substitutions at the intersubunit interfaces of the AMPA receptor subunit GluA2, and confirmed via SDS-PAGE that the majority of these cysteines form intersubunit crosslinks. We tested the functional outcome of crosslinking domains by recording GluA2-mediated currents in reducing and non-reducing conditions. Strong redox-dependent changes in GluA2-mediated currents were observed for cysteine substitutions at the LBD dimer-dimer interface but not at the ATD dimer-dimer interface. We conclude that during gating, LBD dimers either maintain their relative positioning or their relative displacement has no appreciable effect on AMPA receptor function.

## Methods

### Constructs, expression and purification

For crosslinking and Fluorescence-detection Size Exclusion Chromatography (FSEC)^47^ experiments, the full length rat GluA2i (flip) (NP_058957) subunit (also known as GluRBi or GluR2i)^48^,^49^, including the native signal peptide, was subcloned into the pEG vector for expression in baculovirus-transduced HEK293 GnTI^–^ cells^50^. For fluorescence detection and purification purposes, coding sequences for a thrombin cleavage site (GLVPRG), enhanced green fluorescent protein (eGFP)^51^ and the Strep-tag (WSHPQFEK) were introduced at the carboxyl terminus. The point mutation C589A was introduced to reduce non-specific disulfide bond formation^5^. The resulting construct (GluA2_C589A-Thr_) was expressed in HEK293 GnTI^−^ cells.

HEK293 GnTI^–^ cells were harvested 60–96 hours after addition of BacMam P2 virus and collected by a low speed centrifugation (6000 rpm, 10 min). Cells were disrupted using a Misonix Sonicator (18 × 15 s, power level 7) in a buffer containing 150 mM NaCl, 20 mM Tris-HCl (pH 8.0), 1 mM β-mercaptoethanol (βME), 0.8 μM aprotinin, 2 μg/ml leupeptin, 2 μM pepstatin A and 1 mM phenylmethysulfonyl fluoride (25 ml buffer/1L Sf9 cells culture). The homogenate was clarified using a Sorval centrifuge (8000 rpm, 15 min) and crude membranes were collected by ultracentrifugation (Ti45 rotor, 40000 rpm, 1 hour). The membranes were mechanically homogenized and subsequently solubilized for 2 hours in a buffer containing 150 mM NaCl, 20 mM Tris-HCl (pH 8.0), 1 mM βME and 20 mM C_12_M (*n*-dodecyl-β-D-maltopyranoside; 0.25 g of C_12_M/1 g membranes). Insoluble material was removed by ultracentrifugation (Ti45 rotor, 40000 rpm, 40 min) and streptactin resin (0.5–1.0 ml per liter of cells) was added to the supernatant. After binding for 3–18 hours, the protein was eluted with the buffer containing 150 mM NaCl, 20 mM Tris-HCl (pH 8.0), 1 mM βME, 1 mM C_12_M, and 2.5 mM desthiobiotin. Following concentration and thrombin digestion (1:200 mass ratio of thrombin to receptor, 1 hour at 22 °C), the receptor-containing solution was loaded onto a size exclusion chromatography (SEC) Superose 6 column equilibrated in a buffer composed of 150 mM NaCl, 20 mM Tris-HCl (pH 8.0), 2 mM βME, 1 mM C_12_M and 0.01 mg/ml lipid – 3:1:1 POPC:POPE:POPG (1-palmitoyl-2-oleoyl-sn-glycero-3-phosphocholine: 1-palmitoyl-2-oleoyl-sn-glycero-3-phosphoethanolamine, and 1-palmitoyl-2-oleoyl-sn-glycero-3-[phospho-rac-(1-glycerol)]). Peak fractions were pooled and concentrated to ~2 mg/ml for protein crosslinking experiments. All steps were performed at 4 °C unless otherwise noted.

### Cysteine crosslinking

Single cysteine substitutions in GluA2_C589A-Thr_ were introduced using conventional PCR-based methods. Constructs were verified by sequencing over the entire length of the iGluR coding region. The parent (GluA2_C589A-Thr_) or single cysteine substituted constructs in the pEG vector were expressed in HEK293 GnTI^–^ cells and purified as described above. 2 mM dithiothreithol (DTT) as well as 6 mM Glu and 100 μM CTZ (Glu+CTZ) or 6 mM Glu (Glu) or 1 mM NOW (NOW) or 200 μM ZK 200775 (ZK) or no ligands (Apo) were added to the purified protein. Protein was then extensively dialyzed against SEC buffer that did not contain any reducing agent but was supplemented either with 3 mM Glu and 50 μM CTZ (Glu + CTZ) or 3 mM Glu (Glu) or 0.5 mM NOW (NOW) or 100 μM ZK 200775 (ZK) or no ligands (Apo), respectively. For several selected mutants with better protein yield (I209C, V215C, A621C and R628C), we repeated this experiment by supplementing the dialysis buffers with the oxidizing mixture of 25 μM cupric sulfate and 100 μM 1,10-Phenanthroline (Cu:Phen). In the presence of Cu:Phen, the dimeric bands became better resolved for I209C and A621C, while no significant difference was observed for V215C and R628C. A small portion (~10%) of the dialyzed protein was subjected to FSEC (Supplementary Fig. 1). The rest was subjected to denaturing conditions by addition of 6X SDS sample buffer containing 300 mM Tris-Cl (pH 6.8), 12% SDS, 0.6% bromophenol blue and 60% glycerol in the absence (non-reducing condition) or presence (reducing condition) of 100 mM DTT. The protein samples were then run on SDS PAGE gel and protein bands were visualized by Coomassie blue staining.

### Electrophysiology

DNA encoding wild type or cysteine-substituted GluA2 or GluA2_C589A-Thr_ was introduced into a plasmid for expression in eukaryotic cells^49^ that was engineered to produce green fluorescent protein via a downstream internal ribosome entry site^52^. Human embryonic kidney HEK293 cells grown on glass cover slips in 35-mm dishes were transiently transfected with 1–5 μg of plasmid DNA using Lipofectamine 2000 Reagent (Invitrogen). Recordings were made 24 to 96 hours after transfection at room temperature. Currents from whole cells or from outside-out patches, typically held at a –60 mV potential, were recorded using Axopatch 200B amplifier (Molecular Devices, LLC), filtered at 5 kHz and digitized at 10 kHz using low-noise data acquisition system Digidata 1440A and pCLAMP software (Molecular Devices, LLC). The external solution contained (in mM): 140 NaCl, 2.4 KCl, 4 CaCl_2_, 4 MgCl_2_, 10 HEPES pH 7.3 and 10 glucose; 7 mM NaCl was added to the extracellular activating solution containing 1 mM L-glutamate (Glu). The internal solution contained (in mM): 150 CsF, 10 NaCl, 10 EGTA, 20 HEPES pH 7.3. Rapid solution exchange was achieved with a two-barrel theta glass pipette controlled by a piezoelectric translator. Typical 10–90% rise times were 200–300 μs, as measured from junction potentials at the open tip of the patch pipette after recordings. Data analysis was performed using the computer program Origin 9.1.0 (OriginLab Corp.). Recovery from desensitization recorded in two-pulse protocols was fitted with the Hodgkin-Huxley equation^53^: *I* = (*I*_max_^1/*m*^ – (*I*_max_^1/*m*^ – *I*_0_^1/*m*^) × exp(–*t*/*τ*))^*m*^, where *I* is the peak current at a given interpulse interval, *t*, *I*_max_ is the peak current at long interpulse intervals, *I*_0_ is the current at zero time, *τ* is the recovery time constant and *m* is an index that corresponds to the number of kinetically equivalent rate-determining transitions that contribute to the recovery time course.

## Additional Information

**How to cite this article**: Yelshanskaya, M. V. *et al.* Probing Intersubunit Interfaces in AMPA-subtype Ionotropic Glutamate Receptors. *Sci. Rep.* **6**, 19082; doi: 10.1038/srep19082 (2016).

## Supplementary Material

Supplementary Information
