# Supplementary material for: Probing Intersubunit Interfaces in AMPA-subtype Ionotropic Glutamate Receptors
Source: Sci Rep. 2016 Jan 7;6:19082. doi: 10.1038/srep19082 (PMC4703952; doi:10.1038/srep19082)
Supplement: Supplementary Information [file srep19082-s1.doc]

Supplementary Information

(Supplementary Figure 1 and Supplementary Tables 1-3)

Probing Intersubunit Interfaces in AMPA-subtype Ionotropic Glutamate Receptors

Maria V. Yelshanskaya, Kei Saotome, Appu K. Singh and Alexander I. Sobolevsky

**
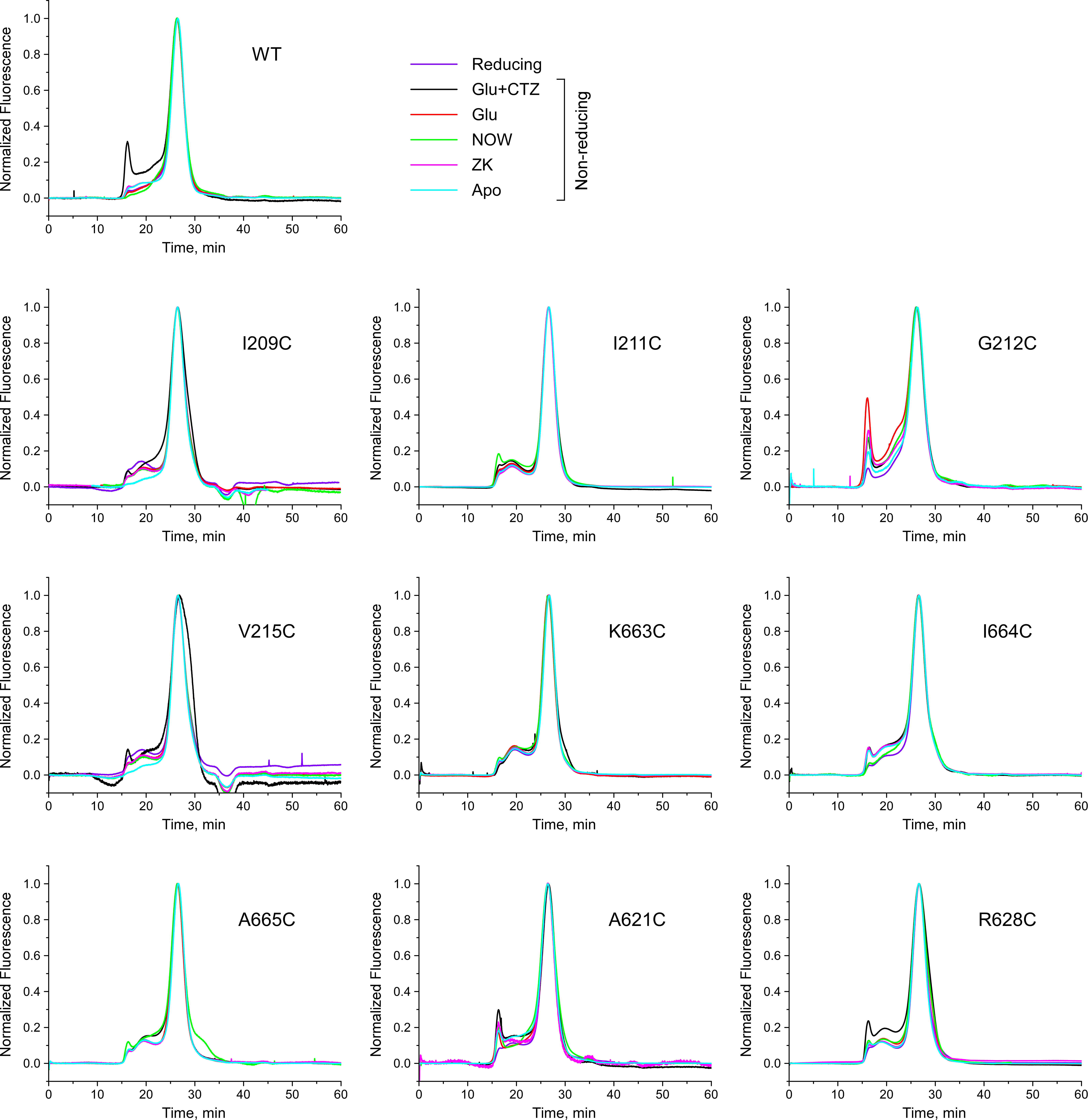
**

**Supplementary Figure 1.** **FSEC analysis of purified cysteine-substituted mutants.** Wild type (WT) and cysteine-substituted GluA2_C589A-Thr_ receptors were purified and kept in reducing conditions (Reducing) or exchanged into non-reducing buffers containing either 3 mM Glu and 50 µM CTZ (Glu+CTZ, favoring the open state) or 3 mM Glu (Glu, favoring the desensitized state) or 500 µM NOW (NOW, favoring the desensitized state) or 100 µM ZK (ZK, favoring the antagonist bound closed state) or no ligands (Apo, favoring the unliganded closed state), loaded onto a Superose 6 SEC column in line with a detector monitoring tryptophan fluorescence. Most FSEC traces demonstrate a single monodisperse peak eluted at a time (0.5 mL/min flow rate) typical for iGluR tetramer. Some peak broadening was observed for several mutants in the presence of NOW or Glu+CTZ (e.g., I209C, V215C, K663C, A665C and R628C) indicating possible aggregation or breakdown of tetramers. Nevertheless, most of the protein remained in the tetrameric state.

**Supplementary Table 1. Effect on activation.**

| Construct | *I*_P_/*I*_P,DTT_ | *n* | *I*_0_/*I*_0,DTT_ | *n* |
| --- | --- | --- | --- | --- |
| WT | 1.04 ± 0.03 | 13 | 1.10 ± 0.11 | 10 |
| WT_C589A-Thr_ | 0.95 ± 0.08 | 10 | 1.08 ± 0.06 | 6 |
| I209C | 1.06 ± 0.12 | 17 | 1.07 ± 0.03 | 8 |
| I211C | 0.91 ± 0.10 | 10 | 0.96 ± 0.09 | 5 |
| G212C | 0.93 ± 0.14 | 8 | 1.09 ± 0.15 | 5 |
| V215C | 0.90 ± 0.07 | 9 | 1.06 ± 0.06 | 7 |
| K663C | 0.96 ± 0.10 | 10 | 0.97 ± 0.11 | 8 |
| I664C | 0.20 ± 0.04 | 12 | 0.28 ± 0.11 | 7 |
| A665C | 0.18 ± 0.03 | 25 | 0.26 ± 0.09 | 7 |
| R628C | 0.80 ± 0.07 | 25 | 0.83 ± 0.07 | 25 |
| A621C | 1.21 ± 0.10 | 7 | 1.07 ± 0.08 | 6 |

The ratios of peak currents amplitudes (*I*_P_/*I*_P,DTT_) and maximal current amplitudes in the continuous presence of CTZ (*I*_0_/*I*_0,DTT_) measured in the absence and presence of DTT. Highlighted are values significantly different from 1; t-Test (p < 0.05). Errors are SEM.

**Supplementary Table 2. Parameters of desensitization.**

|  | Non-reducing | | | | Reducing | | | |
| --- | --- | --- | --- | --- | --- | --- | --- | --- |
|  | Non-desensitized fraction | | Time constant | | Non-desensitized fraction | | Time constant | |
| Construct | *I*_SS_/*I*_0_ | *n* | *τ*_Des_ | *n* | *I*_SS,DTT_/*I*_0,DTT_ | *n* | *τ*_Des,DTT_ | *n* |
| WT | 0.027 ± 0.005 | 10 | 7.1 ± 0.3 | 17 | 0.035 ± 0.007 | 7 | 7.1 ± 0.4 | 14 |
| WT_C589A-Thr_ | 0.032 ± 0.013 | 6 | 7.0 ± 0.4 | 10 | 0.033 ± 0.008 | 7 | 7.2 ± 0.4 | 10 |
| I209C | 0.052 ± 0.012 | 9 | 7.8 ± 0.3 | 19 | 0.059 ± 0.008 | 8 | 7.7 ± 0.3 | 17 |
| I211C | 0.040 ± 0.017 | 5 | 8.3 ± 0.6 | 10 | 0.029 ± 0.010 | 7 | 7.7 ± 0.6 | 10 |
| G212C | 0.080 ± 0.041 | 5 | 6.6 ± 0.3 | 11 | 0.052 ± 0.036 | 3 | 6.5 ± 0.5 | 8 |
| V215C | 0.040 ± 0.013 | 7 | 7.9 ± 0.6 | 12 | 0.050 ± 0.012 | 8 | 8.4 ± 0.8 | 11 |
| K663C | 0.042 ± 0.011 | 8 | 11.7 ± 0.6 | 11 | 0.046 ± 0.018 | 6 | 11.7 ± 0.5 | 10 |
| I664C | 0.041 ± 0.008 | 9 | 4.3 ± 0.3 | 16 | 0.036 ± 0.021 | 5 | 3.5 ± 0.3 | 12 |
| A665C | 0.041 ± 0.007 | 10 | 8.8 ± 0.4 | 32 | 0.031 ± 0.008 | 10 | 8.6 ± 0.4 | 26 |
| R628C | 0.237 ± 0.035 | 29 | 9.1 ± 0.4 | 28 | 0.239 ± 0.033 | 25 | 9.0 ± 0.4 | 24 |
| A621C | 0.053 ± 0.006 | 7 | 6.4 ± 0.3 | 8 | 0.062 ± 0.008 | 4 | 6.8 ± 0.5 | 8 |

The fraction of non-desensitized receptors measured in the absence (*I*_SS_/*I*_0_) or presence (*I*_SS,DTT_/*I*_0,DTT_) of DTT and the time constant of desensitization measured in the absence (*τ*_Des_) or presence (*τ*_Des,DTT_) of DTT. Highlighted are values significantly different from the wild type values; t-Test (p < 0.05). Errors are SEM.

**Supplementary Table 3. Parameters of recovery from desensitization.**

|  | Non-reducing | | | | Reducing | | | |
| --- | --- | --- | --- | --- | --- | --- | --- | --- |
| Construct | *I*_max_ | *τ*_Rec Des_ | *m* | *n* | *I*_max_ | *τ*_Rec Des, DTT_ | *m* | *n* |
| WT | 0.995 ± 0.004 | 13.5 ± 1.0 | 2.70 ± 0.35 | 11 | 0.987 ± 0.010 | 16.8 ± 1.6 | 1.86 ± 0.23 | 8 |
| WT_C589A-Thr_ | 0.900 ± 0.015 | 14.1 ± 2.1 | 4.91 ± 1.19 | 5 | 0.920 ± 0.013 | 14.8 ± 1.5 | 4.55 ± 0.71 | 7 |
| I209C | 0.991 ± 0.012 | 18.7 ± 1.6 | 4.74 ± 0.71 | 8 | 0.994 ± 0.013 | 18.6 ± 2.0 | 4.46 ± 0.88 | 10 |
| I211C | 0.988 ± 0.003 | 10.3 ± 0.7 | 2.54 ± 0.23 | 8 | 0.985 ± 0.005 | 9.2 ± 1.0 | 2.74 ± 0.50 | 9 |
| G212C | 0.967 ± 0.013 | 17.6 ± 1.4 | 4.49 ± 0.65 | 7 | 1.006 ± 0.012 | 17.9 ± 1.4 | 5.04 ± 0.85 | 4 |
| V215C | 0.968 ± 0.009 | 19.2 ± 1.8 | 2.11 ± 0.24 | 7 | 0.971 ± 0.010 | 12.6 ± 1.0 | 4.16 ± 0.70 | 6 |
| K663C | 0.927 ± 0.008 | 12.5 ± 1.0 | 3.55 ± 0.59 | 6 | 0.928 ± 0.009 | 16.2 ± 1.5 | 2.00 ± 0.30 | 6 |
| I664C | 0.963 ± 0.011 | 56.7 ± 4.7 | 3.98 ± 0.59 | 13 | 0.944 ± 0.011 | 17.8 ± 1.3 | 3.54 ± 0.42 | 16 |
| A665C | 0.928 ± 0.016 | 15.8 ± 1.9 | 3.12 ± 0.57 | 6 | 0.975 ± 0.060 | 156 ± 7 | 1.42 ± 0.07 | 17 |
| R628C | 0.972 ± 0.006 | 19.2 ± 0.9 | 1.87 ± 0.05 | 22 | 0.962 ± 0.006 | 19.9 ± 0.9 | 1.71 ± 0.06 | 18 |
| A621C | 0.995 ± 0.017 | 15.7 ± 2.4 | 3.67 ± 0.71 | 5 | 1.047 ± 0.020 | 14.4 ± 1.7 | 3.70 ± 0.68 | 4 |

The parameters of Equation 1 fitting: the normalized peak current at long interpulse intervals (*I*_max_), the time constant of recovery from desensitization (*τ*_Rec Des_ or *τ*_Rec Des, DTT_) and the *m* index in the absence (Non-reducing) and presence (Reducing) of DTT. Highlighted are the values of the time constant of recovery from desensitization that show 3-fold and 10-fold differences in reducing and non-reducing conditions for I664C and A665C, respectively. Note the values of the *m* index display significant variation for reasons that require further investigation. Errors are SEM.
